# Study protocol for the Screen-Free Time with Friends Feasibility Trial

**DOI:** 10.1186/s40814-024-01462-y

**Published:** 2024-02-19

**Authors:** Sarah Overgaard Sørensen, Kristian Traberg Larsen, Teresa Victoria Høy, Anders Blædel Gottlieb Hansen, Russell Jago, Peter Lund Kristensen, Mette Toftager, Anders Grøntved, Anne Kær Gejl

**Affiliations:** 1 Research Unit for Exercise Epidemiology, Department of Sports Science and Clinical Biomechanics, University of Southern Denmark, Odense, Denmark; 2 National Institute of Public Health, University of Southern Denmark, Copenhagen, Denmark; 3 Center for Clinical Research and Prevention, Frederiksberg Hospital, Frederiksberg, Denmark; 4 Population Health Sciences, Bristol Medical School, University of Bristol, Bristol, UK; 5 NIHR Bristol Biomedical Research Centre, University Hospitals Bristol and Weston NHS Foundation Trust and University of Bristol, Bristol, UK; 6 Research Unit for Active Living, Department of Sports Science and Clinical Biomechanics, University of Southern Denmark, Odense, Denmark

**Keywords:** Screen time, Time with friends, Feasibility, Acceptability, Compliance

## Abstract

**Background:**

Children are spending less leisure time with their friends in person and an increasing amount of time with digital screens. These changes may negatively affect children’s physical and mental health. The Screen**-**Free Time with Friends Feasibility Trial will test the feasibility, including acceptability and compliance, of an intervention designed to reduce screen media usage and encourage physical interaction with friends during leisure time in 9–11-year-old children.

**Methods:**

A non-randomized single-group feasibility trial will be conducted from March to October 2023 including approximately 75 children (aged 9–11 years) and 75 parents (at least 1 per child) from 3 different schools recruited from 3 different municipalities in Denmark. The Screen-Free Time with Friends intervention is a multicomponent intervention targeting families, afterschool clubs, and local communities. It has been developed using a systematic process guided by the Medical Research Council UK’s framework for developing and evaluating complex interventions. With a systems perspective in mind, the intervention and implementation approach has been designed to facilitate adaptation to the specific needs of diverse local communities while maintaining the core components of the intervention.

Feasibility and acceptability of the intervention will be assessed during the intervention using process evaluation inspired by the RE-AIM framework including questionnaires and interviews with the municipality project managers, research team members, local ambassadors and stakeholders, parents and school, and afterschool club personnel. In addition, participation, recruitment, retention rate, and compliance to the outcome measurements will be investigated and presented.

**Discussion:**

The trial will investigate the feasibility and acceptability of the Screen-Free Time with Friends intervention, the recruitment strategy, and the planned outcome measurements. This feasibility study will investigate necessary refinements before the implementation of the intervention program in a larger cluster randomized controlled trial to evaluate its impact.

Trial registration.

ClinicalTrials.gov, ID: NCT05480085. Registered 29 July 2022. https://clinicaltrials.gov/ct2/show/NCT05480085?cond=Screen+free+time+with+friends&draw=2&rank=1

**Supplementary Information:**

The online version contains supplementary material available at 10.1186/s40814-024-01462-y.

## Background

Screen media devices are ubiquitous in modern society and children frequently spend their leisure time engaged in screen use. In a Danish context, 47% of 10–11-year-old boys and 29% of girls have problematic screen media use (defined as more than 4 h/day with screens during leisure time) on weekends, while on weekdays, the numbers are 35% for boys and 22% for girls [1]. Important possible consequence of excessive screen media use in children during leisure is the displacement of face-to-face time with friends and family and less engagement in physical behaviors [2, 3]. Irrespective of the reason, a decrease in face-to-face social engagement among children may have negative consequences for their development and wellbeing [4]. In Denmark, the proportion of children and adolescents spending time with friends outside of school has been decline [5, 6]. There has also been a decline over the past decade in attendance in afterschool programs in Denmark [6]. The trend of children and adolescents increasingly spending less face-to-face time with their peers during leisure time is also evident in other countries [7].

Since spending time alone after school is closely linked to high amounts of screen media use [8] and may synergistically contribute to unhealthy child development [9], interventions that encourage face-to-face interactions with friends after school could effectively reduce children’s recreational screen media use, increase well-being, promote physical activity, and support their natural development as they transition into adolescence. The Screen-Free Time with Friends Feasibility Trial is a study designed to inform the development of an extracurricular school-based intervention program aiming to promote more time face to face with friends and reduce recreational screen media use after school and during weekends among children aged 9–11 years. The specific objectives of the Screen-Free Time with Friends Feasibility Trial are to examine the feasibility of the intervention content, recruitment process, and data collection plan to identify elements that require additional refinement. Furthermore, the feasibility trial aims to obtain baseline and follow-up data on the planned full trial primary outcome to determine the required sample size for the full trial.

## Methods

### Study design and participants

The Screen-Free Time with Friends Feasibility Trial will be conducted as a non-randomized single-group feasibility trial from March to October 2023 (https://clinicaltrials.gov ID: NCT05480085). The feasibility trial aims to include approximately 75 children (aged 9–11 years), 75 parents (at least one per child), and school and afterschool personnel from 3rd-grade school classes at three different schools recruited from three different municipalities in Denmark. Furthermore, 15–18 local stakeholders from each of the three local areas surrounding the schools will be included. The local stakeholders will be described in more detail in the section about local workshops. A formal sample size calculation is not conducted as the study will not incorporate hypothesis testing. This approach is in line with the guidelines of the CONSORTS extension for pilot and feasibility trials [10]. However, the goal was to recruit approximately 25 children and parents from one or more classes at each school, as well as 15–18 local stakeholders in each municipality, as we determined that this sample size would be representative of the overall population. Moreover, this sample size aligns with the recommendations for sample sizes for pilot studies [11]. Additionally, existing literature indicates that achieving saturation in qualitative data can be accomplished with a participant range of 9 to 17 [12].

The feasibility trial has been approved by the Research Ethics Committee at the University of Southern Denmark (22/29625) and will be conducted in accordance with the principles of the Declaration of Helsinki. Parents will be required to provide consent on behalf of themselves and their child prior to enrolling in the project. This study protocol is developed based on the SPIRIT 2013 checklist for study protocols of clinical trials (additional file 1).

### Recruitment

**Fig. 1:**
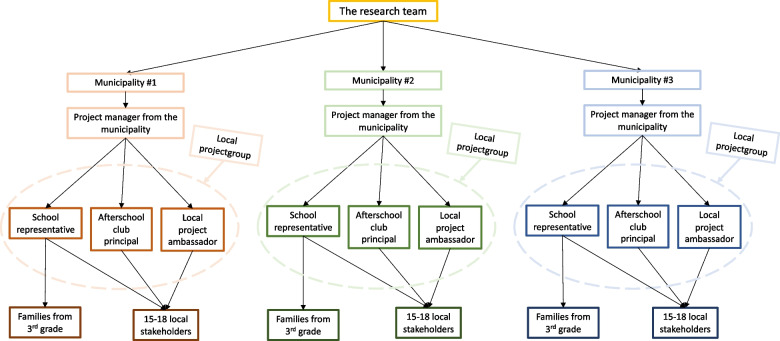
Recruitment process for the Screen-Free Time with Friends Feasibility Trial

The recruitment process in the Screen-Free Time with Friends Feasibility Trial, shown in Fig. 1, will be conducted from June 2022 until the end of May 2023. Prior to the recruitment, the research team will conduct a prioritized list of possible municipalities. Eligible municipalities will be contacted until three municipalities are interested in participating in the study. The interested municipalities will be asked to appoint a project manager from the municipality (i.e., municipality project manager) who will be invited to a meeting with representatives from the research team. Here, the research team will elaborate on the project, and the parties will match expectations. If the municipality agrees to participate in the project, the project manager will be asked to recruit one school and the related afterschool club from the municipality. In the full trial, we expect that the project manager will be recruiting six schools, which will be randomized by the research group into an intervention and control group. As a part of the intervention, the project manager will be asked to establish a local project group comprising a school representative, the afterschool club principal, and a local ambassador. The local ambassador represents the local community and should have insight into the local children’s leisure time activities and possibilities. Following the establishment of the local project group, they will be invited to a meeting with a representative from the research team and the municipality project manager. At this meeting, they will get detailed information about the project and receive oral and written information about their role in the project. For the full trial, the establishment of a local project group will only occur in the communities where the school is randomized to the intervention group.

The school representative will assist the research team in the recruitment of children and families from 3rd grade at the school. The families will be invited to a meeting where the project and its methods will be explained fully, and the families will receive written information about the project. After the meeting, the families will have time to consider whether they wish to participate in the study. The local project group will be responsible for the recruitment of 15–18 local stakeholders relevant to children’s leisure time.

### Inclusion and exclusion criteria

School and afterschool club eligibility will be assessed based on the following criteria: (1) the school has a related afterschool club in a distance that allows for self-transportation (walking or cycling distance) (assessed by the municipality); (2) the afterschool club must have potential for improvement, e.g., increasing the participation rate (assessed by the municipality); and (3) the afterschool club principal and personnel must be willing to develop the afterschool club by participating in a training program. The goal is to include a representative sample of children and parents from the participating 3rd grades, and, thus, there will be no further inclusion or exclusion criteria according to the participating families.

### Intervention

The development and evaluation of the Screen-Free Time with Friends intervention follow the Medical Research Council (MRC) framework for developing and evaluating complex interventions [13]. The intervention is based on a thorough and systematic development process [14], with continuous revisiting of the core elements of the MRC framework. Our aim was to develop an intervention that considers several of the multiple factors that impact children’s behavior during leisure time by adopting a systemic approach [15]. Throughout the development process, numerous stakeholders and experts have contributed. One example of stakeholder involvement is a participatory system mapping process that engaged a group of national stakeholders with diverse organizational affiliations and perspectives related to children’s leisure time [16]. The outcome of this process was a more comprehensive understanding of the system, which established the basis for determining the core intervention components. Additionally, stakeholders representing our target groups have participated in developing the specific intervention content and provided crucial feedback on feasibility and acceptability. Finally, we have developed an implementation strategy that aims to increase the intervention’s sustainability as well as feasibility in different contexts by allowing the intervention to adapt to the distinct needs of local communities while preserving the intervention’s core components. This approach has recently been recommended in a perspective article describing a new context-specific approach for designing and analyzing interventions [17].

**Fig. 2:**
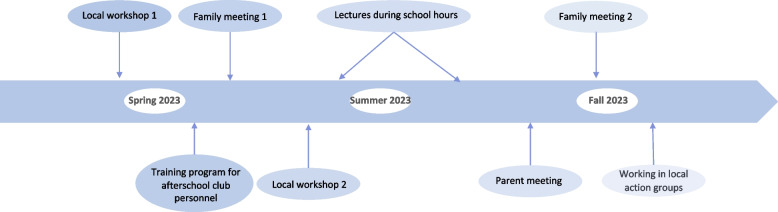
Timeline for the Screen-Free Time with Friends Feasibility Trial intervention

This work has resulted in an intervention that comprises three core components, targeting the following: (1) parents and children, (2) afterschool club personnel, and (3) local stakeholders. The intervention will start in spring 2023 where the children are in 3rd grade and end in the fall 2023 where the children are attending 4th grade. The timeline and intervention components can be seen in Fig. 2 and will be described in more details below.

### Family meetings and lectures during school hours

Parents and peers play important roles in shaping children’s behavior, and it is important to consider both when trying to promote behavior change in children [18]. Studies also indicate that increased parental involvement is a successful approach in interventions aimed at decreasing children’s screen time [19–22]. As such, a key aspect of the intervention is to engage parents and peers in addressing screen media habits and face-to-face interactions during leisure time within both peer groups and families.

#### Family meetings

Two “family meetings” will be held for parents outside of school hours, which will include education, sharing of experiences, and group discussions. The meetings will focus on school-class culture related to the use of screen media and how parents can encourage face-to-face interaction among their children during leisure time. In addition to the two family meetings, parents will engage in a short exercise focusing on screen media dilemmas pertinent to the particular age group. The teacher will conduct this exercise in the beginning of 4th grade to boost parents’ motivation to participate in the second family meeting. The exercise also aims to inspire parents to collaborate on developing shared screen media principles for their children in the class. The school representative from the local project group will be responsible for planning and conducting the family meetings as well as for informing the 4th-grade teachers about the exercise. While the research team will provide general guidelines and materials for the meetings and exercise, they will not intervene in the practical aspects surrounding the meetings.

The first family meeting will concentrate on leisure time activities, while the second meeting focuses on screen media habits among the children and their families. In both meetings, parents will receive information to enhance their overall understanding of the two subjects and establish a foundation for subsequent discussions. An important aim of the first meeting is to increase the parents’ awareness of the afterschool club including activities and policies. Attendance for children at the first meeting is optional. The overall aim of the second meeting is to promote parent-to-parent communication and to provide them with knowledge and strategies to establish and sustain healthy screen media habits for their children. For example, there will be a focus on the family’s digital everyday life, including screen time, how digital interruptions are experienced from a child’s perspective, and how to create good agreements in the family. During the second meeting, children will participate and share information to inspire and encourage the parents to discuss and reflect on screen media behaviors. The main aim of involving children is to enhance parents’ understanding of their children’s screen media usage and preferences and promote communication within families and among parents. The children’s contribution to the meeting will be prepared through a series of lectures during school hours.

#### Lectures during school hours

Three lectures will be conducted during school hours, in which children will discuss the advantages and disadvantages of using screens, as well as the classroom culture associated with screen usage. The lectures will also cover the differences between face-to-face interactions and online communication. The teacher will facilitate the lectures using the materials provided by the research team. However, the teacher will have the flexibility to use other relevant material related to screen usage. The school representative from the local project group will be responsible for informing the teachers involved about the lectures, forwarding the suggested material provided by the research team, and ensuring that the lectures are completed. The aims of the lectures are to encourage children to reflect on their own screen media habits and to initiate discussions about the classroom culture associated with screen use and whether any changes are necessary. For example, data on children’s screen use may be gathered and used in class to raise awareness of their habits. The information obtained from these lectures may be shared by the children and/or teacher during the family meetings as mentioned above.

### Afterschool club

In order to increase children’s participation in the afterschool club, the personnel will take part in a training program with the aim of increasing the number of 4th-grade children who attend the club regularly. The program consists of three workshops and is facilitated by a Danish organization, Ungdomsringen, a Danish nongovernmental organization (NGO) promoting healthy youth relationships by supporting the interests of Danish youth centers. The sole role of the research team is to facilitate contact between the afterschool club principal and Ungdomsringen who will have the primary responsibility of planning and conducting the three workshops. During the first workshop, the participants creates a “status report” on their afterschool club using a screening model, with a focus on the five themes identified by the project’s stakeholders to maintain children’s engagement with afterschool clubs: (1) relationship between the afterschool club personnel and the children; (2) collaboration among the fourth-grade afterschool club, the third-grade afterschool club, and the school; (3) communication of the afterschool club activities and pedagogic standpoint, (4) screen usage within the afterschool club; and (5) children’s codetermination. This approach helps identify what actions the club is currently undertaking within these five themes and what they aim to do more of or differently in the future. As a part of the second workshop, the participants will be asked to select one to three areas of focus that they want to develop, formulate goals, and plan mini actions to achieve these goals. For instance, a goal can be to increase the collaboration with the school by organizing visits and conducting activities in the afterschool club for the children during school hours in collaboration with the teachers. From the second to the third workshop, the personnel will gain experience with the actions, which will be evaluated in the third workshop. Ultimately, this will result in a final plan for actions valid for the specific club in order to increase the number of 4th-grade children attending the club on a daily basis. The inclusion of the afterschool club personnel in identifying areas of focus in the specific afterschool club is aligned with the recommendations for developing context-specific interventions [17].

### Local workshops

As a part of the intervention, 15–18 local community stakeholders will be invited to participate in two workshops. The local ambassador will be responsible for planning the workshops, whereas the local project group will share the responsibility of recruiting local stakeholders. These stakeholders may represent parents, school teachers, afterschool club personnel, local politicians, and others relevant to children’s leisure time (local representatives from, e.g., sports clubs, library, church). The teachers and personnel from the afterschool club who are already participating in the project should be invited to attend the local workshops, and they will receive economic compensation for their participation in the workshops. The research team will facilitate the two workshops and provide the local project groups with information and invitations for the workshops, but they will not interfere with the practical planning of the workshops or recruitment of stakeholders. The overall aim of the workshops is to establish a collective agreement for action among stakeholders, encouraging the implementation of locally customized actions. The workshops will build on the methodology of group model building, which uses inputs from multiple perspectives to gain a shared understanding [23]. Group model building has been found to improve problem understanding, enhance stakeholder engagement and confidence in systems thinking, and promote consensus for action among stakeholders [24–26].

Throughout the two workshops, the research team will involve the local stakeholders in systems thinking activities, which will result in a visual representation of the system using causal loop diagrams. The purpose of these activities is to help stakeholders to understand the dynamics of the system and promote change at the systems level through shared understanding [27]. Using the causal loop diagrams, stakeholders will identify existing actions within the system and prioritize the most important areas for action. Local actions will then be outlined, and action groups will be created based on interest, willingness, and power to make changes in the specific area.

### Measurements

The following measurements will be conducted to evaluate the feasibility of the intervention, the recruitment process, and the measurement methods. Some of the measurements will be conducted before and after the intervention, while others will be carried out at specific timepoints or continuously during the intervention period (Table 1).

**Table 1:** Assessments in the Screen-Free Time with Friends Feasibility Trial

Assessments	Study period				
	*Enrolment*	*Baseline*	*Continuously during the intervention*	*After completion of specific components*	*6-month follow-up*
	*Fall 2022 to spring 2023*	*Spring 2023*	*Spring 2023 to fall 2023*		*Fall 2023*
Eligibility screening of school and afterschool club	x				
*Feasibility of the intervention*		x		x	
*Participant recruitment and retention rate*		x			x
*Compliance to leisure time activities measurement*		x			x
*Compliance to device-based screen media use measurement*			x		
*Compliance to subjective screen media use measurement*		x			x
*Compliance to device-based physical activity measurement*		x			x
*Compliance to wellbeing measurement*		x			x
*Compliance to child questionnaire*		x			x
*Compliance to parent questionnaire*		x			x

### Primary outcome

#### Feasibility of the intervention

The feasibility of the Screen-Free Time with Friends intervention will be investigated by examining the acceptability and compliance to the intervention assessed both prior to and at specific time points during the intervention period. The feasibility of the intervention will be evaluated using process evaluation inspired by the RE-AIM framework [28, 29], which has been widely used when evaluating public health interventions [30]. The RE-AIM framework focuses on five dimensions: Reach, Effectiveness, Adoption, Implementation and Maintenance [28]. In the current study, the effectiveness dimension will be evaluated based on changes in behavioral determinants, as the feasibility trial does not aim to investigate the effects of the intervention. As shown in Table 2, the process evaluation will involve objective measurements (physical activity, height, and weight) among the children and the use of questionnaires and interviews with the municipality project managers, research team members, local ambassadors and stakeholders, parents, and school and afterschool club personal. Height and weight will be measured to the nearest 0.5 cm and 0.1 kg, respectively, at the schools at baseline and follow-up using standard methods. The measurements will be conducted in a private room by a researcher of same sex. The interviews will be conducted as phone, individual, or focus-group interviews. The aim is to interview all municipality project managers and local ambassadors at the three municipalities, 1–2 schoolteachers and afterschool club personal from each school, and a representative sample of parents from the included school classes. School and afterschool club personnel as well as parents will be recruited based on a purposeful sampling strategy. The information obtained during the process evaluation will be used to evaluate what components of the intervention that works and what components require further refinement before the definitive trial. Furthermore, the process evaluation will be used to assess the participants attitude to the measurements described below.

**Table 2:** Overview of the process evaluation inspired by the RE-AIM framework

Dimension	Definition	Outcome measure	Data source and time frame
Reach	The representativeness and characteristics of participating children compared to children in the target group	Participation recruitment and retention rate in the study and characteristics of participating children compared to children in the target group	Previous studies conducted among Danish children Questionnaires answered by parents and children at baseline Anthropometric (height and weight) and physical activity measurements at baseline
Effectiveness	Factors influencing change in behavioral outcomes including knowledge and engagement	Determinants of behavior based on the Theoretical Domain Framework (e.g., knowledge, skills, beliefs about capabilities) among parents Knowledge and engagement among local workshop participants	Questionnaire inspired by the Theoretical Domain Framework answered by parents at baseline and after completing the family meetings Questionnaire inspired by COMPACT Stakeholder-driven Community Diffusion Survey answered by local stakeholders before and after the local workshops
Adoption	The commitment of participating municipalities, schools, afterschool clubs, and families regarding their decision to be a part of the intervention and factors influencing that decision	Factors influencing the participant’s commitment and engagement in the intervention	Interviews conducted with the municipality project manager, local ambassador, school- and afterschool club personnel, and selected parents after completion of the intervention components
Implementation	The extent to which schools, afterschool clubs, and local community implemented the components as intended and adaptations made to the components	Number of intervention components implemented Adaptation to the intervention components	Questionnaires and interviews with intervention delivers after completion of the components
Maintenance	The extent to which intervention components have the ability to become an integrated part of daily practice	Factors influencing maintenance	Questionnaire and interviews with intervention delivers after completion of the components

### Secondary outcomes

#### Participant recruitment and retention rate

Participant recruitment rate will be assessed at baseline as the proportion of invited municipalities, schools, afterschool clubs, children, parents, and local stakeholders included in the study. Furthermore, retention rate will be assessed at follow-up as the proportion of municipalities, schools, afterschool clubs, children, parents, and local stakeholders completing the study.

#### Compliance to leisure time activities measurement

Children’s time spent face-to-face with peers, the primary outcome for the full trial, will be parent reported three times a week for 3 weeks at the beginning and end of the intervention period. Parents will be asked questions about their children’s activities after school and during weekends. The questions will cover the amount of time their child spent face to face with friends or family, as well as the time they spend in afterschool club or sport clubs. Compliance to the leisure time activities measurement will be assessed as proportion of parent’s answering these questions.

#### Compliance to device-based screen media use measurements

Children and parents’ smartphone time will be assessed objectively continuously during the intervention by the Ethica application (https://ethicadata.com) installed on the participant’s devices. Ethica is an application that records time when the smartphone’s screen turns on or off. Compliance to the device-based screen media use measurement will be assessed by the proportion of children and parents with data on device-based measured smartphone use during the intervention period. Furthermore, the amount of data of device based screen use will be reported.

#### Compliance to subjective screen media use measurements

In addition to the device-based measurement of children’s smartphone use, their total screen media use (including other devices than smartphones) will be parent reported. In connection with the leisure time activity questionnaire, parents will receive questions about their child’s use of screen media devices alone and physically with friends three times per week for 3 weeks at baseline and 6-month follow-up. Compliance to the subjective screen media use measurement will be assessed by the proportion of parents answering the questions about their child’s screen media use in the beginning and end of the intervention period.

#### Compliance to device-based physical activity measurement

Physical activity will be assessed by Axivity AX3 triaxial accelerometers (https://axivity.com/product/ax3). At school, the research team will provide the children with accelerometers and instruct them to wear the device at the thigh using two adhesive patch 24 h/day for seven consecutive days at baseline and 6-month follow-up [31]. Based on the three-axis accelerometry data, time spent within physical activity intensity domains (sedentary, light, moderate, and vigorous) and distinct activity types (sitting or lying, moving, standing, biking, running, and walking) will be measured. These latter activity types can be determined with very high accuracy in children [32].

In addition to the physical activity measurement, the Axivity AX3 triaxial accelerometers will be used to assess children’s sleep.

Compliance to the Axivity AX3 triaxial accelerometers, measurement will be assessed as the proportion of children with at least three valid weekdays and at least one valid weekend day at baseline and follow-up. A valid day will be defined as a measurement day with less than 10% non-wear time during leisure time. Non-wear will be identified as described in Rasmussen et al. (2020) [33].

The OmGui software (https://github.com/digitalinteraction/openmovement/wiki/AX3-GUI) will be used to prepare the accelerometers and download data after the measurement periods.

#### Compliance to wellbeing measurement

The children’s wellbeing will be assessed by self-report using the Danish version of the KIDSCREENS-27 questionnaire at baseline and 6-month follow-up. The KIDSCREEN-27 is a widely used questionnaire translated to at least 38 languages containing 27 items focusing on five dimensions: physical wellbeing, psychological wellbeing, parent relations and autonomy, social support and peers, and school [34]. The items will be answered on a 5-point Likert scale (1 = never; 5 = always). Previous international studies have demonstrated that the KIDSCREEN-27 has a high test–retest reliability (*ICC*: 0.56–0.77) and acceptable internal consistency (Cronbach’s alpha: 0.80–0.84) among children aged 8–18 years [34]. Furthermore, the KIDSCREEN-27 has shown good and satisfactory convergent, known groups and criterion validity in an international study [34]. Compliance to the wellbeing measurement will be assessed as proportion of children answering the KIDSCREEN-27 at baseline and follow-up.

#### Compliance to child questionnaire

In addition to completing the validated KIDSCREEN-27 questionnaire, children will be asked to answer a short questionnaire developed by the research team that focuses on their wellbeing, social relations, and screen media culture in the class and family.

The child questionnaire includes the Cantril’s ladder, which is an 11-point scale that is commonly utilized to evaluate subjective wellbeing [35]. The ladder has been used extensively in both Danish and international studies that investigate children’s health and wellbeing [6, 36]. Children will be asked to rate their current position on the ladder, which will be presented to them as follows: “Here is a ladder. The top of the ladder ‘10’ is the best possible life for you and the bottom ‘0’ is the worst possible life for you. In general, where on the ladder do you feel you stand at the moment?”.

Based on questions from the child questionnaire, social network analysis will be employed to examine the children’s social relationships within the class. For instance, children will be asked who they prefer to spend their leisure time with. There will be no restriction on the number of friends children can note from the same class. Social relations will be evaluated as the number of times the child nominates others (out-degree) and is nominated by others (in-degree). This method has previously been used in studies investigating the association between social relations and physical activity among children aged 5–14 years in school and afterschool clubs [37–39].

Compliance to the child questionnaire will be assessed by the proportion of children answering the questionnaire at baseline and follow-up.

#### Compliance to objective height and weight measurements

As mentioned above, children’s height and weight will be measured at the schools at baseline and follow-up. Compliance will be reported as the proportion of children completing these measurements.

#### Compliance to parent questionnaire

At baseline and 6-month follow-up, parents will be asked to fill out a questionnaire about background information and their child’s and family’s physical activity and screen media habits and addiction. Questions about the screen media habits and screen media culture in the family, household constellation, parents’ educational level according to the International Standard Classification of Education (ISCED), and working status were adopted from the SCREENS-Q [40]. The SCREENS-Q which is developed to assess children’s screen media habits and possible related correlates have previously been shown to have moderate to high test–retest reliability of all evaluated items [40]. Besides, the sociodemographic background collected using the SCREENS-Q parents will be asked to report the birthday, gender, and ethnicity of themselves and their child.

The questions adopted from the SCREENS-Q include the smartphone addiction scale — short version (SAS-SV) consisting of 10 items rated on a 6-point Likert scale (strongly disagree to strongly agree) to assess the level of risk of smartphone addiction among parents, where higher scores indicate a higher risk of smartphone addiction [41]. Furthermore, the parent questionnaire will include the Problematic Media Use Measure Short Form (PMUM-SF) to assess children’s screen media addiction [42]. The PMUM-SF is developed based on the criteria for Internet gaming disorder suggested by the American Psychiatric Association [43] and consists of nine items with responses on a 5-point Likert scale (1 = never; 5 = always) [42]. A higher score indicates a higher level of screen media addiction. When answering the items, the parents must consider the past month and their child’s overall screen media use [42]. A previous study showed good reliability (Cronbach’s alpha = 0.93) and convergent and incremental validity of the PMUM-SF in a sample of mothers of children aged 4–11 years [42].

Compliance to the parent questionnaire will be assessed as the proportion of parents answering the questionnaire at baseline and 6-month follow-up.

### Data management

The interview data will be kept on secure servers located at the University of Southern Denmark. Ethica and SurveyXact will be used to collect questionnaire data, which will then be extracted and securely stored on servers. Similarly, the data collected from device-based measurements will be extracted from the devices and stored securely on servers. The data will be stored in its raw form, and no statistical analysis will be conducted before the feasibility trial is complete.

### Analysis

Interviews will be audiotape recorded, transcribed verbatim, and analyzed using qualitative content analyses. In the presentation of the results from the interviews, data will be anonymized. Descriptive analysis of the process evaluation questionnaires and interviews will be performed to determine the utilization and attitude of the municipality project manager, research team members, local ambassador, parents, and school and afterschool club personnel. The need for adjustments of the recruitment process, intervention content, and data collection plan will be based on the information obtained from these qualitative analyses and a judgement of the research team. Nevertheless, the primary criteria of success will be as follows:

1. The municipalities have to establish a local project group comprising a school representative, the afterschool club principal, and a local ambassador.
2. All intervention activities have to be completed.
3. A total of 85% of the parents have to answer the leisure time activity questionnaire, the primary outcome for the full trial, three times/week for at least 1 week.

Participation recruitment, retention rate, and compliance assessment will be presented. All recruited participants will be included in the analyses.

Baseline and follow-up data on children’s time spend face to face with friends after school and during weekends will be used to assess the necessary sample size to obtain a power of 80% to detect a minimal meaningful difference with alpha = 0.05 in the definitive full cluster-randomized controlled trial.

Qualitative analyses will be conducted in NVivo, and quantitative analyses will be conducted using STATA version 17. The results will be published in scientific journals.

## Discussion

The current protocol describes in detail a non-randomized single-group feasibility trial that aims to examine the feasibility of an extracurricular school-based intervention program aiming to promote time spent face to face with friends and reduce screen media use during leisure time among Danish children aged 9–11 years.

Evidence has shown that both physical activity and screen media use are associated with time spent with friends [44, 45]. Furthermore, face-to-face interactions with peers are positively associated with children’s wellbeing, while screen media use is negatively associated with wellbeing [46]. Therefore, encouraging face-to-face interaction with friends could potentially be an effective approach to increase physical activity levels and wellbeing among children. However, to our knowledge, no previous experimental research has explored this particular approach. It is especially crucial to develop this type of intervention in modern society as the number of children spending time with friends after school is decreasing [5]. This means that an increasing number of children are spending time alone at home after school, which in today’s screen-saturated environment progressively is associated with screen time and physical inactivity [8]. This may in turn have serious implications for the overall wellbeing of children [46].

Our goal is to create an intervention that takes a systemic perspective by considering multiple factors that influence children’s behavior during leisure time. A systems perspective recognizes that behavior is influenced by a complex web of interrelated factors, including individual factors (such as attitudes, beliefs, and emotions), social factors (such as peer pressure, family dynamics, and cultural norms), and environmental factors (such as physical surroundings, infrastructure, and policies) [15]. By acknowledging interventions as events within a system, it becomes apparent that any change resulting from the intervention can have ripple effects throughout the system. These effects may be positive or negative, intended or unintended, and may impact different parts of the system in various ways. Therefore, considering interventions as events within systems allows for a more comprehensive and holistic approach to intervention design and implementation, potentially leading to a more efficient and sustainable intervention [13, 47, 48].

The completion of the Screen-Free Time with Friends Feasibility Trial will enable us to evaluate the intervention’s feasibility and acceptance and subsequently refine it accordingly. We will gather insights on the intervention from various groups involved in the study through interviews, providing diverse perspectives on the intervention. Nonetheless, as the feasibility study only includes one school from each municipality and does not involve a control group, certain aspects of the recruitment process will not be assessed. However, the current study will inform the development and refinement of an intervention, with the goal of promoting habitual physical activity and well-being. If effective and in a longer perspective, the most promising elements of the intervention could have the potential to be implemented on a larger scale with a broader target group in focus.

### Supplementary Information

**Additional file 1.** SPIRIT 2013 Checklist: Recommended items to address in a clinical trial protocol and related documents.

## Data Availability

Not applicable.

## Abbreviations

MRC: Medical Research Council
SAS-SV: Smartphone addiction scale-short version
PMUM-SF: Problematic Media Use Measure Short Form
ISCED: International Standard Classification of Education
